# Photoactivation of LOV domains with chemiluminescence†

**DOI:** 10.1039/d3sc04815b

**Published:** 2023-12-11

**Authors:** Yuhao Ji, Ali Heidari, Brice Nzigou Mombo, Seraphine V. Wegner

**Affiliations:** a Institute of Physiological Chemistry and Pathobiochemistry, University of Münster 48149 Münster Germany wegnerse@uni-muenster.de

## Abstract

Optogenetics has opened new possibilities in the remote control of diverse cellular functions with high spatiotemporal precision using light. However, delivering light to optically non-transparent systems remains a challenge. Here, we describe the photoactivation of light-oxygen-voltage-sensing domains (LOV domains) with *in situ* generated light from a chemiluminescence reaction between luminol and H_2_O_2_. This activation is possible due to the spectral overlap between the blue chemiluminescence emission and the absorption bands of the flavin chromophore in LOV domains. All four LOV domain proteins with diverse backgrounds and structures (iLID, BcLOV4, nMagHigh/pMagHigh, and VVDHigh) were photoactivated by chemiluminescence as demonstrated using a bead aggregation assay. The photoactivation with chemiluminescence required a critical light-output below which the LOV domains reversed back to their dark state with protein characteristic kinetics. Furthermore, spatially confined chemiluminescence produced inside giant unilamellar vesicles (GUVs) was able to photoactivate proteins both on the membrane and in solution, leading to the recruitment of the corresponding proteins to the GUV membrane. Finally, we showed that reactive oxygen species produced by neutrophil like cells can be converted into sufficient chemiluminescence to recruit the photoswitchable protein BcLOV4-mCherry from solution to the cell membrane. The findings highlight the utility of chemiluminescence as an endogenous light source for optogenetic applications, offering new possibilities for studying cellular processes in optically non-transparent systems.

## Abstract 2

We describe the photoactivation of light-oxygen-voltage-sensing domains (LOV domains) with *in situ* generated blue light from a chemiluminescence reaction, opening new possibilities for optogenetic tools in optically non-transparent systems.

## Graphical abstract

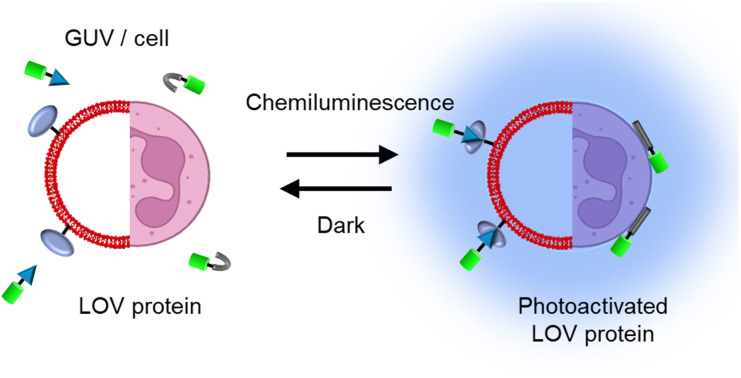

## Introduction

Optogenetics has revolutionized the manipulation of cellular functions with light through the introduction of photo-sensory domains into proteins.^1^ The photoregulation comes with high spatial and temporal precision, does not interfere with other cellular processes and allows dynamic switching on and off functions simply with visible light. Various optogenetic tools have been used for perturbing diverse cellular processes *in vitro* or *in vivo* including but not limited to activation/deactivation of specific signalling networks,^2,3^ transcription-translation,^4–6^ RNA/gene editing,^7,8^ organismal patterning,^9,10^ tissue engineering^11^ and organ formation.^12^

A major hurdle in the field is the difficulty of delivering light to tissues and cells in optically non-transparent systems due to high photon scattering and low penetration.^13^ This has limited most studies to cell culture, transparent organisms (*e.g. Drosophila*, zebra fish and *C. elegans*) and peripheral body parts. In a few examples of deep tissue applications in mice exogenous devices such as optical fibres were implanted as light guides, which are highly invasive.^14^ Recent studies have shown that it is possible to photoactivate proteins with endogenous light from bioluminescence reactions^15–21^ and from upconversion nanoparticles.^22,23^ However, the former approach requires a genetically coded catalyst – luciferase – and the latter approach is still by the penetration of near-infrared photons and thermal effects. In contrast, luminol-based chemiluminescence has been exploited to generate endogenous light relying on reactive oxygen species (ROS) and catalyst present in microenvironments at sites of inflammation.^24–26^

**Fig. 1:**
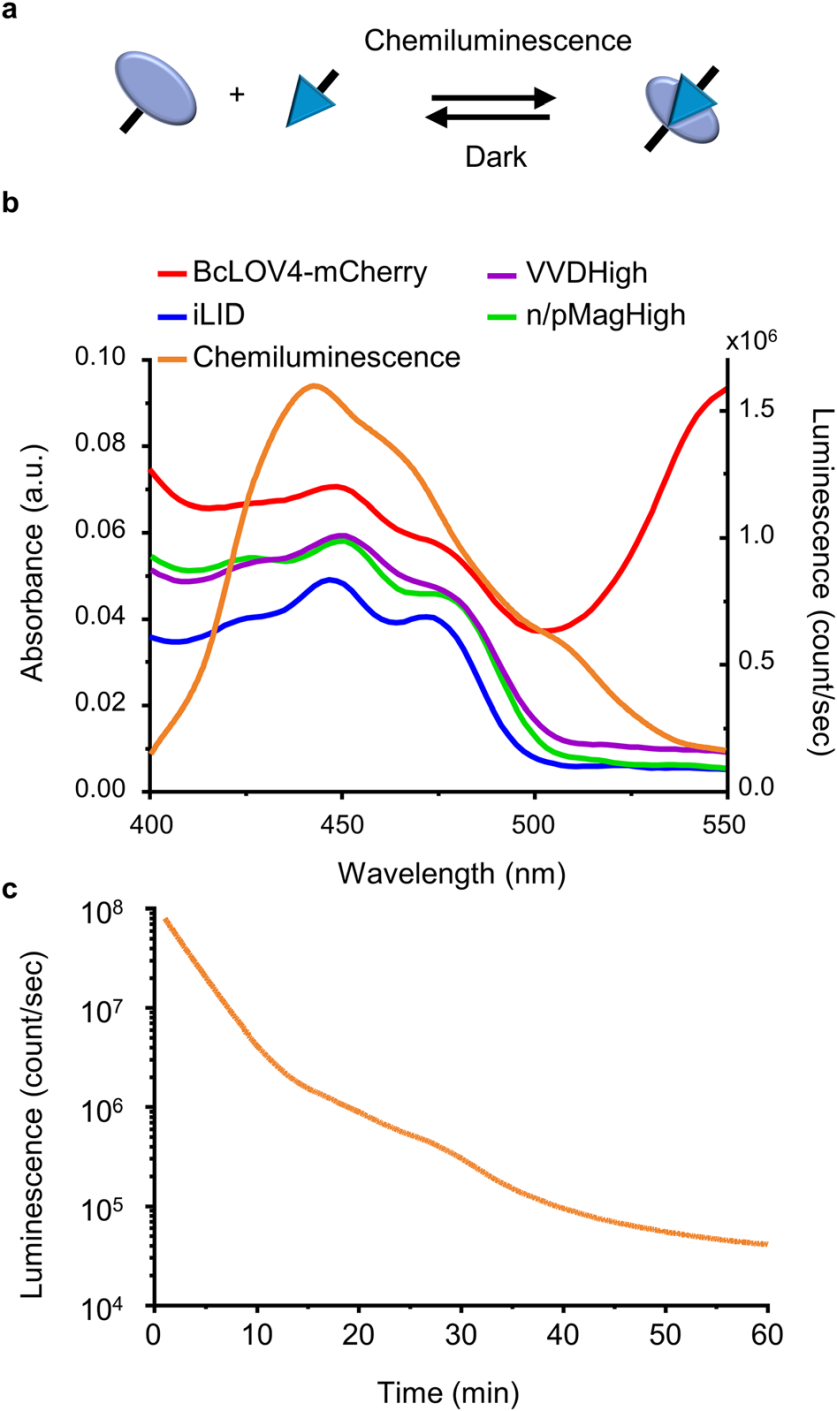
(a) Scheme showing LOV domains are activated with chemiluminescence resulting in protein–protein interactions and reversion in the dark. (b) Overlap of the absorbance spectra of different LOV domain-based proteins and chemiluminescence emission spectrum from the HRP-catalysed reaction between luminol and H_2_O_2_. (c) Kinetics of the chemiluminescence reaction (200 μM luminol, 500 μM H_2_O_2_, and 0.6 U per mL HRP).

Here, we investigate the use of chemiluminescence as an alternative endogenous light source to excite photoswitchable proteins with light-oxygen-voltage-sensing (LOV) domains (Fig. 1a). In a variety of *in vitro* and *in cellulo* assays, we demonstrate that chemiluminescence from the widely known reaction between luminol and H_2_O_2_ is able to activate a repertoire of LOV domain proteins.

## Results and discussion

### Spectral match of LOV domains and chemiluminescence

For this study, we selected four LOV domains proteins:^27^ BcLOV4, which is derived from *Botrytis cinerea* and aggregates with itself under blue light;^28^ iLID, which is derived from the AsLOV2 domain of *Avena sativa* and binds to Nano under blue light;^29^ and VVDHigh,^30^ as well as nMagHigh/pMagHigh^31^ which are derived from *Neurospora crassa* and homo- and hetero- dimerize, respectively, under blue light. All of these LOV domains have a flavin chromophore (flavin adenine dinucleotide (FAD) or flavin mononucleotide (FMN))^27^ and form a covalent bond between the chromophore and a reactive cysteine residue in the protein. Yet, these proteins differ in their mode of action (homodimerizers: BcLOV4 and VVDHigh; heterodimerizers: iLID & Nano and nMagHigh & pMagHigh) and the reversion times in the dark (BcLOV4 *t*_1/2_ = 18.5 s,^28^ VVDHigh *t*_1/2_ = 4.7 h,^30^ iLID & Nano *t*_1/2_ = 20 s,^29^ and nMagHigh & pMagHigh *t*_1/2_ = 4.7 h).^31^ All these photoswitchable proteins containing LOV domains absorb light in the range of 400–500 nm due to their flavin cofactor (Fig. 1b). These absorption peaks overlap with the blue light chemiluminescence emission from the horseradish peroxidase (HRP)-catalysed reaction between luminol and H_2_O_2_. This chemiluminescence reaction generates high photon counts of >10^5^ counts per s for approximately 30 min (Fig. 1c) compared to the background levels of 10^3^ counts per s (Fig. S1†). The reaction kinetics and the photo-counts can be adjusted by changing the concentrations of the reactants with considerable photon production for less than 10 min to hours (Fig. S2†).

### Dynamic colloidal self-assembly built on the photoactivation of LOV domains with chemiluminescence

**Fig. 2:**
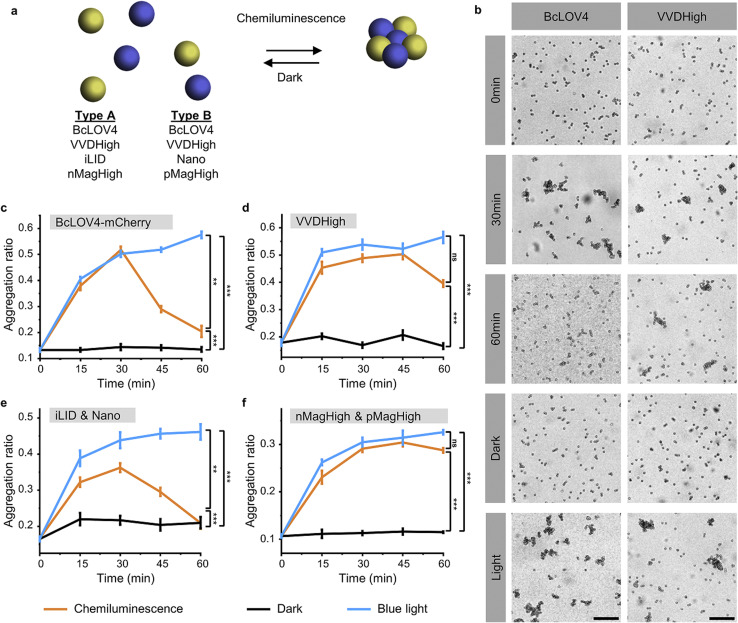
(a) In the bead aggregation assay beads functionalized with complementary photoswitchable proteins aggregate upon photoactivation with chemiluminescence. The bead aggregation reverses once the chemiluminescence output is low with kinetics determines by the dark reversion rate of the photoswitchable protein–protein interaction. (b) Aggregation of 2 μm polystyrene beads coated with BcLOV4-mCherry or VVDHigh with a chemiluminescence reaction (200 μM luminol, 500 μM H_2_O_2_, and 0.6 U per mL HRP) at different time points. Beads under continuous blue illumination and in the dark for 30 min as positive and negative controls, respectively. Scale bars are 30 μm. (c)–(f) Aggregation ratios of beads functionalized with different LOV domain proteins and their interaction partners with chemiluminescence, under blue light and in the dark.

To investigate and compare the photoactivation of the different LOV domains with chemiluminescence, we employed the bead aggregation assay. Here, the light-activated interactions of the different LOV proteins that are immobilized on beads reversibly result in the aggregation of the particles (Fig. 2a). In particular, the beads can aggregate due to the light-activated homo-oligomerization of BcLOV4 and VVDHigh^30^ as well as the hetero-dimerization of iLID with Nano and nMagHigh with pMagHigh.^32^ To achieve this, we immobilized the respective proteins, bearing His6-tags, onto 2 μm diameter polystyrene beads functionalized with Ni^2+^-NTA (nitrilotriacetic acid) groups, as previously described.^30,32^ In the case of hetero-dimerizing pairs, we mixed equal numbers of beads bearing complementary interaction partners. Subsequently, we incubated the different bead samples under three conditions: a chemiluminescence reaction solution (containing luminol, H_2_O_2_, and HRP), under blue light illumination (positive control), or in the dark (negative control).

Through bright field microscopy, we observed that BcLOV4-coated beads formed distinct bead aggregates under chemiluminescence conditions within 30 min, akin to the positive control samples exposed to blue light (Fig. 2b). In contrast, the beads remained dispersed in the dark. Similarly, VVDHigh, iLID/Nano and nMagHigh/pMagHigh beads also aggregated under chemiluminescence conditions as they did under blue light but remained as single beads in the dark (Fig. 2b and S3–6†). These results demonstrated that all four photoswitchable protein interactions could be activated through chemiluminescence.

The timeline of bead aggregation is governed by the interplay between the kinetics of the chemiluminescence reaction, the strength of light-activated protein–protein interactions, and the dark reversion kinetics exhibited by the different LOV domains. In order to elucidate this dynamic process, we conducted quantitative analyses of aggregate formation and disassembly mediated by various photoswitchable proteins. To this end, we quantified the formation and disassembly kinetics of the aggregates mediated by different photoswitchable proteins by imaging large areas of the samples (1.11 mm^2^) and computing from them the aggregation ratio (the aggregation ratio is equal to the area occupied by aggregates larger than 10 beads divided by the area occupied by all beads) (Fig. 2c–f and S3–6†).

For BcLOV4-coated beads under chemiluminescence conditions, we observed the formation of distinct bead aggregates within 30 min and a majority of them disassembled into mostly single beads within 60 min comparable to the sample kept in the dark (Fig. 2c and S3†). The dynamic changes in colloidal aggregation observed under chemiluminescence agreed with the temporal decrease in photon counts from the reaction (Fig. 1c). Initially, when the photon output was high (>10^5^ counts per s) for the first 30 min, BcLOV4 was activated, resulting in aggregate formation. Subsequently, as the photon production from the chemiluminescence reaction diminished over time, the fast dark reversion of BcLOV4 became dominant, leading to the coupled disassembly of the aggregates. It should be noted that during the reversion processes still photon counts above the background (<10^3^ counts per s, Fig. S1†) were measured but they were not sufficient to counteract the reversion reaction. Analogous to the BcLOV4-coated beads, the iLID & Nano mediated aggregates showed a temporal pattern of formation and disassembly over the course of 60 min (Fig. 2e and S5†).

In contrast, the beads functionalized with VVDHigh exhibited a different time profile in their aggregation behaviour under the same chemiluminescence reaction conditions (Fig. 2d and S4†). Within 15 min, these beads assembled into large aggregates, which remained stable for up to 45 min, similar to the samples exposed to blue light. Only after 60 min did we observe a slight disassembly of these aggregates. Similar to the VVDHigh-coated beads, nMagHigh & pMagHigh-coated beads aggregated that did not reverse within 60 min (Fig. 2f and S4†). These observed differences in disassembly kinetics align with the faster dark reversion kinetics observed for BcLOV4 and iLID, in contrast to the slower kinetics observed for VVDHigh, nMagHigh and pMagHigh.

It is important to highlight that in all instances, the beads exhibited comparable levels of aggregation when exposed to chemiluminescence or direct blue illumination after 30 min. This significant observation underscores the complete photoactivation of these LOV domains through the internally generated luminescent light. Furthermore, we noted that the bead aggregation ratio for photoswitchable proteins with homophilic interactions was higher than proteins with heterophilic interactions, possibly because the number of beads of one type is twice as large in the homophilic aggregation compared with the heterophilic one. In addition, the aggregation only occurred in the presence of all chemiluminescence reagents (luminol, H_2_O_2_ and HRP), not if one of the reaction components was missing (Fig. S7 and 8†). Collectively, these findings provide compelling evidence for the successful photoactivation of these four distinct LOV domains using chemiluminescence as an alternative light source.

Typically, the photoactivation of LOV domains is monitored by observing the decrease in absorbance of the chromophore following blue light illumination.^27^ However, this approach was not viable in the presence of the chemiluminescence reaction components, as they also absorb light in a similar range. In previous studies, we successfully demonstrated the photoactivation of iLID binding to Nano using a competitive fluorescence polarization assay.^33^ Unfortunately, this assay is not transferable to the other proteins investigated here. Therefore, the bead aggregation assay serves as an experimental setup to investigate and compare different LOV domains with a microscopic readout.

### Local photon production inside GUVs

**Fig. 3:**
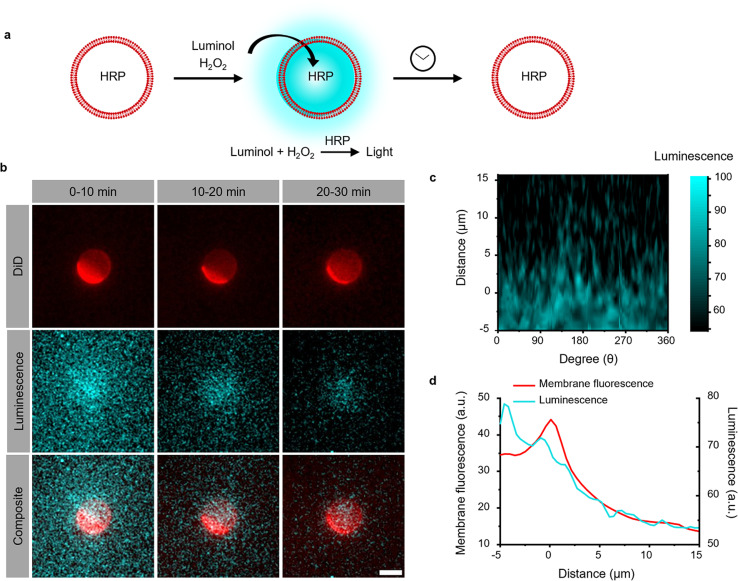
(a) Chemiluminescence is produced inside the GUVs used as a HRP loaded reactor upon addition of the permeable substrates luminol and H_2_O_2_. (b) Luminescence and fluorescence microscopy images of GUV (membrane labelled with DiD shown in red) generating chemiluminescence (shown in cyan) inside. Scale bar is 10 μm. (c) Chemiluminescence intensity from the GUV after polar transformation in the time interval 0–10 min. 0 μm was defined as the edge of the GUV in the DiD channel. (d) Spatial distribution of luminescence and membrane fluorescence. Chemiluminescence intensity was observable outside the membrane but rapidly decreased at distances >5 μm.

The photoactivation with chemiluminescence opens the possibility to trigger LOV domains at the site and the time of photon emission. To demonstrate this internal spatiotemporal control, we employed giant unilamellar vesicles (GUVs) as reactors capable of producing intravesicular chemiluminescence. Notably, the photons emitted from chemiluminescence reaction can pass the optically transparent lipid membrane of the GUVs and can potentially excite photoswitchable proteins located outside the vesicle (Fig. 3a).

To test this hypothesis, we encapsulated the catalyst for the luminescence reactions, HRP, within the GUVs and added the membrane permeable substrates luminol and H_2_O_2_ externally. Chemiluminescence measurements of solutions containing these GUVs showed photon production but not if only the surrounding buffer without GUVs was used (Fig. S9†). The kinetics of intravesicular chemiluminescence exhibited initial delays and prolonged emission time, contrasting with the reaction kinetics in bulk solution (Fig. 1c).^33^ This difference arises due to the additional diffusion of luminol and H_2_O_2_ across the GUV membrane. This production of intravesicular chemiluminescence was visualized using luminescence/fluorescence microscopy, revealing the presence of a chemiluminescent halo surrounding the GUVs, which decays over time (Fig. 3b). Luminescence was strongest inside the GUV and showed a glowing halo around the GUV with a thickness of less than 5 μm (Fig. 3c and d). As luminescence imaging requires long integration time (here 10 min), we tried to suppress the movement of the GUV by modifying its membrane with 0.5 mol% biotin-DOPE and immobilizing it onto a supported lipid bilayer composed of DOPC and 0.5 mol% streptavidin–biotin-DOPE.

### Dynamic activation of iLID on the membrane of GUVs with localized chemiluminescence production

**Fig. 4:**
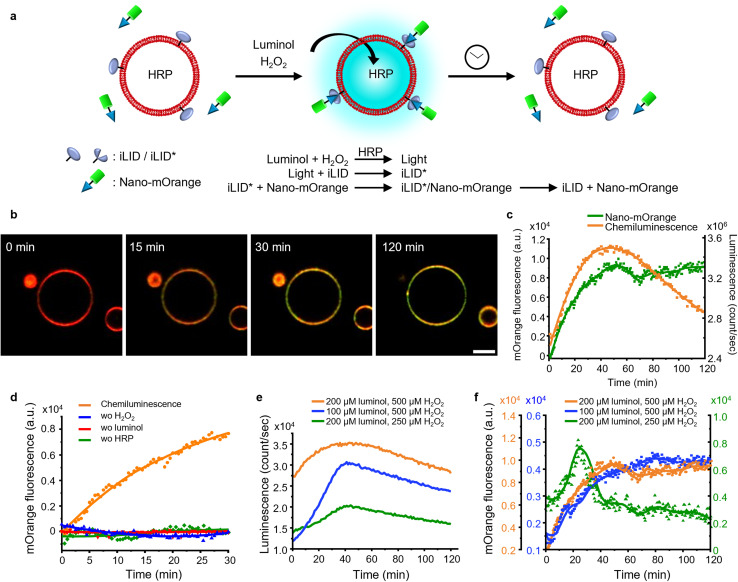
(a) Schematic representation of Nano-mOrange recruitment on iLID functionalized GUV membranes with intravesicular chemiluminescence. (b) Confocal fluorescence microscopy images of Nano-mOrange (shown in green) recruitment kinetics on the GUV membrane (DiD staining, shown in red). Scale bar is 10 μm. Also see Movie 1.† (c) Quantification of Nano-mOrange on the GUV membrane in (b) and chemiluminescence emission dynamics in GUVs as measured with a plate reader. (d) Negative controls for (b), where each of the reagents or catalyst for the chemiluminescence reaction was left out. (e) Comparison of chemiluminescence emission dynamics in GUVs with different substrate concentrations. (f) Comparison of Nano-mOrange recruitment on the GUV membrane under the conditions in (e).

Firstly, we investigated the capability of internal chemiluminescence to directly activate a LOV domain protein that was immobilized on the outer membrane of GUVs (Fig. 4a). To accomplish this, we employed a streptavidin linker to immobilize biotinylated iLID on the leaflet of the GUVs^34^ (POPC, 2 mol% biotin-DOPE, labeled with 1 mol% DiD, encapsulating, and 0.6 U per mL HRP). Simultaneously, Nano-mOrange (200 nM) was introduced into the exterior of the iLID-functionalized GUVs, along with intravesicular chemiluminescence reagents (200 μM luminol and 500 μM H_2_O_2_). Over time, we observed an enrichment of mOrange fluorescence at the periphery of these GUVs, due to the binding of Nano to the photoactivated iLID (Fig. 4b, S10 and Movie 1†).

In this system, the photon counts from the chemiluminescence reaction increased for approximately 40 min and later, as the reactants were consumed, the intensity of the chemiluminescence decreased (Fig. 4c). Quantitative analysis of mOrange fluorescence on the GUVs revealed an increases recruitment of the protein for one hour, followed by a stable plateau that persisted for up to two hours (Fig. 4c). The initial recruitment of Nano-mOrange to the membrane exhibited a delay compared to chemiluminescence production, suggesting a sequential order of events. The dissociation of Nano-mOrange was not observed even when the chemiluminescence decreased and remained at the plateau for up to 2 h (Fig. 4c) as the chemiluminescence production was still high enough to sustain iLID activation.

The recruitment of Nano-mOrange was attributed to the chemiluminescence occurring within the GUVs, as there was no such recruitment observed when any of the three reaction components (luminol, H_2_O_2_, or HRP) were absent from the system (Fig. 4d and S11†). Additionally, in a positive control experiment involving direct and localized blue illumination, Nano-mOrange was rapidly recruited to the membrane of iLID-functionalized GUVs and gradually diffused to non-illuminated regions (Fig. S12a†). This recruitment process was reversible in the dark within 10 min (Fig. S12b†).

To modulate the recruitment of Nano-mOrange to the GUV membrane and enable reversible binding, we modified the intravesicular chemiluminescence production profile. Specifically, we reduced the concentrations of either luminol (100 μM) or H_2_O_2_ (250 μM). In both cases, we observed a similar bell-shaped luminescence emission timeline (Fig. 4e). However, the overall photon production was significantly lower compared to the previous experimental condition (200 μM luminol and 500 μM H_2_O_2_). Notably, when reducing H_2_O_2_ concentrations, the chemiluminescence production reached its lowest point, suggesting that H_2_O_2_ acts as the limiting reagent for intravesicular cellular chemiluminescence generation.

Reducing the concentration of luminol resulted in a decreased recruitment of Nano-mOrange to the membrane, reaching its peak at 60 min. However, even in this scenario, the binding between Nano-mOrange and iLID was not reversible within the duration of the experiment (Fig. 4f and S13†). When the H_2_O_2_ concentration was lowered, accompanied by the lowest photon counts, the binding of Nano-mOrange was significantly increased and reached its peak at 30 min (Fig. 4f and S14†). Subsequently, the interaction between mOrange-Nano and iLID reversed after 45 min from the initiation of the chemiluminescence reaction. This experiment confirmed that intravesicular chemiluminescence can activate iLID, and the reversibility can be modulated by changing the efficiency of chemiluminescence.

### Photoactivation of BcLOV4-mCherry in solution to GUVs with internal chemiluminescence

**Fig. 5:**
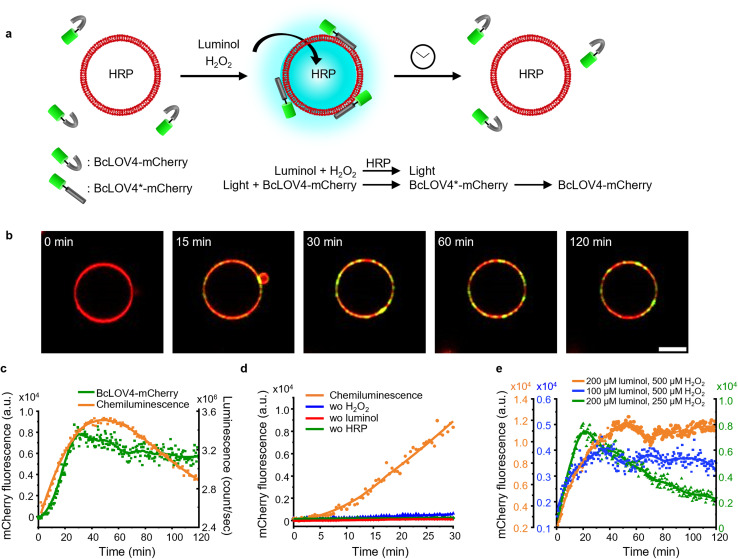
(a) Schematic representation of BcLOV4-mCherry recruitment on GUV membranes with intravesicular chemiluminescence. (b) Confocal fluorescence microscopy images of BcLOV4-mCherry (shown in green) recruitment kinetics on the GUV membrane (DiD staining, shown in red). Scale bar is 10 μm. Also see Movie 2.† (c) Quantification of BcLOV4-mCherry on the GUV membrane in (b) and chemiluminescence emission dynamics in GUVs as measured with a plate reader. (d) Negative controls for (b) where each of the reagents or catalyst for the chemiluminescence reaction was left out. (e) Comparison of BcLOV4-mCherry recruitment on the GUV membrane with different substate concentrations as in Fig. 4e.

Next, we investigated if it is possible to recruit a LOV domain protein from solution to the membrane of a chemiluminescence-producing GUV (Fig. 5a). To accomplish this, we added external BcLOV4-mCherry, which is known to bind to negatively charged lipid membranes when exposed to blue light,^28^ to chemiluminescence-producing GUVs (POPC with 30 mol% POPG and 1 mol% DiD). Following the addition of luminol (200 μM) and H_2_O_2_ (500 μM), we observed an increase in mCherry fluorescence on the GUV membrane (Fig. 5b, c, S15 and Movie 2†). The mCherry fluorescence steadily increased on the GUV membrane, reaching its maximum after 30 min and remaining relatively stable for up to 2 h (Fig. 5c). Throughout the 2 h imaging period, BcLOV4-mCherry did not exhibit noticeable dissociation from the GUV membrane, even as the intravesicular chemiluminescence decreased after approximately 1 h. BcLOV4-mCherry failed to bind to the GUVs if any of the components required for the chemiluminescence reaction were omitted (Fig. 5d and S16†). As a positive control, BcLOV4-mCherry rapidly recruited to the illuminated area of the GUV membrane and subsequently diffused across the entire GUV within less than 2 min (Fig. S17a†). This recruitment of BcLOV4-mCherry was reversed within 10 min in the dark (Fig. S17b†).

Subsequently, we repeated BcLOV4-mCherry recruitment experiments with lowered luminol and H_2_O_2_ concentrations (Fig. 5e, S18 and 19†). In each case, the mCherry fluorescence on the GUV membrane reached its peak approximately 20–30 min after initiating the chemiluminescence reaction. However, we observed dissociation of BcLOV4-mCherry from the membrane only in the sample with the reduced H_2_O_2_ concentration (Fig. 5e and S19†), and the sample with the lowered luminol concentration showed no noticeable decrease in mCherry fluorescence on the membrane for up to 2 h (Fig. 5e and S18†). These findings were consistent with the chemiluminescence production, as decreased H_2_O_2_ led to weaker luminescence that decreases below the threshold for BcLOV4 activation after 30 min. Overall, these results demonstrated that the intravesicular chemiluminescence effectively triggered the binding of BcLOV4 to the membrane, similar to the iLID–Nano interaction, and this binding could be modulated through photon production within the GUVs.

### BcLOV4-mCherry recruitment to ROS producing cells with chemiluminescence

**Fig. 6:**
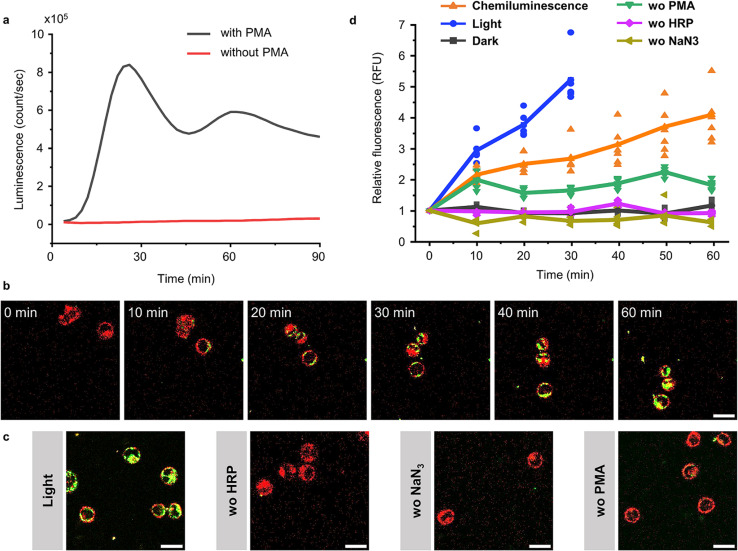
BcLOV4-mCherry recruitment to dHL 60 cells through chemiluminescence. (a) Chemiluminescence generated by dHL60 cells after stimulation with 1 μM PMA in the presence of 2 U per mL HRP, 10 μM L-012 and 1 mM NaN_3_. (b) Confocal fluorescence microscopy images of BcLOV4-mCherry (50 nM, shown in green) recruitment to dHL 60 cells (labelled with DiL-Cell Tracker, shown in red) producing chemiluminescence as in (a). Scale bar is 20 μm. Also see Movie 3.† (c) Positive controls under blue illumination (0.5% 488 nm laser) after 30 min and negative controls in the absence of HRP, NaN_3_ or PMA after 60 min. Scale bar is 20 μm. (d) Quantification of BcLOV4-mCherry on the cell membrane. For each condition 6 cells (shown as individual points) from two technical replicates were analysed.

Although elevated concentrations of ROS are often considered to be harmful to cells, they are an important component of our innate immune defence.^35^ Chemiluminescence based on luminol and its derivatives has been widely employed for detecting ROS production during inflammation *in vitro* and *in vivo*.^36^ Therefore, we investigated if it is possible to activate BcLOV4 with the chemiluminescence produced from ROS and thereby recruit BcLOV4 to ROS producing cells. For this purpose, we selected the human leukaemia cell line HL 60,^37^ which can be differentiated into granulocyte-like dHL 60 cells with DMSO.^38^ These differentiated cells can generate a respiratory burst upon stimulation with the protein kinase C (PKC) activator phorbol myristate acetate (PMA).^39^ In our experiments, we optimized the differentiation of the HL 60 cells as 1 day in the presence of 1.25% (v/v) DMSO (Fig. S20†) and confirmed the conversion into dHL 60 cells using the surface marker for differentiated neutrophils, CD11b,^37^ by fluorescence flow cytometry (Fig. S21†). We observed bright chemiluminescence when stimulating dHL60 cells with 1 μM PMA in the presence of 2 U per mL HRP as the chemiluminescence catalyst, L-012 (ref. 40) used as an alternative to luminol with higher chemiluminescence under cell culture conditions (Fig. S22†) and NaN_3_ as the catalyse inhibitor to avoid H_2_O_2_ decomposition. The chemiluminescence peaked 30 min after the addition of PMA and lasted for at least 90 min (Fig. 6a and S23†).

After having established a ROS coupled chemiluminescence generation by a neutrophil-like cell type, we evaluated the activation and membrane recruitment of BcLOV4-mCherry based on this chemiluminescence. Upon stimulation of the dHL 60 cells (membrane stained with DiD, shown in red) in the presence of soluble BcLOV4-mCherry (shown in green), we found a gradual increase in mCherry fluorescence on the cell membranes for up to 1 h (Fig. 6b and Movie 3†). Consistent with this observation, we also saw an increase in BcLOV4-mCherry fluorescence in the positive control under blue light illumination (Fig. 6c and S24a†), but not in the negative control samples in the dark (Fig. S24b†) and absence of HRP (chemiluminescence catalyst), NaN_3_ (the inhibitor of catalase) or PMA (trigger for respiratory burst) in the dark (Fig. 6b and S25†).

Quantitative analysis of mCherry fluorescence on the cell membrane showed that membrane binding of BcLOV4-mCherry activated by chemiluminescence was slower than direct blue light illumination but reached similar levels over the course of 60 min (Fig. 6d). The sample lacking PMA activation showed a somewhat higher background, which might be due to some leaky ROS production. These results demonstrated that ROS concentration produced by neutrophil like cells can be used to generate sufficient chemiluminescence for BcLOV4-mCherry. This activity could potentially be used to target BcLOV4 conjugated species to sites of inflammation.

## Conclusion

The photoactivation of various LOV domains presented herein is achieved not by external blue light but through *in situ* generated chemiluminescence, as a novel avenue for photoactivation. We showed that this chemiluminescence based activation is compatible with proteins in solution, in cell mimetic vesicles and cell culture. Such LOV domains, widely prevalent in plants, algae, and fungi, assume pivotal roles in light response regulation within these organisms and are the basis of numerous optogenetic tools.^27^ Considering the central role of LOV domains in optogenetics, alternative photoactivation mechanisms with luminescence are highly attractive for locations difficult to reach optically and circumvent background scattering.

In fact, chemiluminescence has been widely used in bioimaging^41^ and immunoassay^42^ because of its high sensitivity, fast reaction kinetics and the readily available reagents. Notably, chemiluminescence's dependence on ROS makes it highly specific for sites of disease, including tumors,^43^ inflammation^44^ and cancers.^45^ In contrast to other possible luminescence reactions that necessitate genetic modifications^46^ or the delivery of nanoscale materials,^47^ the chemiluminescence reaction only requires simple reagents. In this work, we employed a classical chemiluminescence-reaction based on the HRP-catalysed oxidation of luminol with H_2_O_2_. Yet, many other chemiluminescence reactions with different reagents for adjustable photon yield^40^ and kinetics^48^ as well as without enzyme catalysts^49^ exist and are potential alternatives to the here-used chemiluminescence reaction. Furthermore, apart from ROS-rich environments, other chemiluminescence reactions allow for specifically imaging a variety of abnormal physiological environments, including nitroreductase activity in tumor microenvironments and hypoxia,^50^ endogenous activity of β-galactosidase in tumors,^51^ intracellular H_2_S,^52^ and amyloid beta species *in vivo*.^53^ In this respect, the chemiluminescence activation of LOV domains combined with existing chemiluminescence bioimaging setups may open a way not just to visualize but functionally target sites of disease with optogenetics.

## Experimental

### Materials

POPC (1-palmitoyl-2-oleoyl-*glycero*-3-phosphocholine, Cat# 850457), POPG (1-palmitoyl-2-oleoyl-*sn-glycero*-3-phospho-(1′-*rac*-glycerol) sodium salt, Cat# 840457), biotin-PE (1,2-dioleoyl-*sn-glycero*-3-phosphoethanolamine-*N*-(cap biotinyl) (sodium salt), and Cat# 870273) in chloroform were purchased from Avanti Polar Lipids. Streptavidin (Cat# 434301), the membrane dye DiD (1,1′-dioctadecyl-3,3,3′,3′-tetramethylindodicarbocyanine, 4-chlorobenzenesulfonate salt, Cat# D7757), Vybrant™ DiD Cell-Labeling Solution (Cat# V22887) and RPMI 1640 Medium were purchased from Thermo Fisher Scientific. Chemicals and enzymes including luminol (Cat# 123072), L-012 (8-amino-5-chloro-2,3-dihydro-7-phenyl-pyrido[3,4-*d*]pyridazine-1,4-dione, sodium salt) (Cat# SML2236) and phorbol-12-myristate-13-acetate (Cat# P8139) were purchased from Sigma-Aldrich. Ni^2+^-NTA functionalized polystyrene beads (2 μm) were purchased from Micromod Partikeltechnologie GmbH. PE labelled IgG1 mouse anti-human CD11b (eBioscience, Cat# 12-0018) was used. Confocal microscopy experiments with GUVs were performed in 18-well μ-slide glass bottom chambers (Cat# 80827) from ibidi. Bead aggregation experiments were performed using an 8 well μ-slide (Cat# 80826) from ibidi. Buffers and aqueous solutions were prepared with Milli-Q grade water.

### Plasmids and proteins

pQE-80L iLID (C530M) and pQE-80L MBP-SspB Nano were gifts from Brian Kuhlman (Addgene # 60408 and 60409,^29^ respectively). His6_BcLOV4_mCherry_BamUK and His6_BcLOV4_C292A_mCherry_BamUK were gifts from Brian Chow (Addgene # 114596 and 119761, respectively). nMagHigh, pMagHigh^32^ and VVDHigh^30^ were inserted into pET21b between the NdeI and XhoI cutting sites to include a C-terminal His6-tag. The mOrange-GGS was inserted into the pQE-80L MBP-SspB Nano plasmid after the BamHI cutting site to yield His6-MBP-TEV-mOrange-Nano.^54^ AviTag (GLNDIFEAQKIEWHE) was inserted between EcoRI and BamHI restriction cutting sites into the pQE-80L iLID (C530M) plasmid to yield AviTag-iLID.^34^ All proteins were expressed and purified as previously reported.

### Absorbance and chemiluminescence measurements

Absorbance and chemiluminescence measurements were performed with a multimode plate reader (Spark, Tecan Life Science) using transparent and white bottom white plates, respectively. For absorbance measurements of 200 μL of protein solution kept in the dark were used. The chemiluminescence spectrum of the luminol–H_2_O_2_–HRP reaction was acquired after mixing 0.6 U per mL HRP, 200 μM luminol and 500 μM H_2_O_2_ in buffer A (10 mM Tris pH 7.4 and 250 mM NaCl). In chemiluminescence emission in solution and of samples with enzyme loaded GUVs were measured with emission filters from 400 nm to 500 nm. The data from the beginning 5 min were not analysed due to fluctuation after addition of chemiluminescence reagents. For the comparison of chemiluminescence from GUVs and in bulk solution, GUVs were washed three times after preparation and allowed to settle down. The luminescence of the buffer on top and the GUVs at the bottom of the sample was measured after addition of the reagents.

### Bead aggregation assay

2 μm polystyrene beads functionalized with Ni^2+^-NTA groups were purchased as a water suspension (50 mg mL^−1^, 1.2 × 10^10^ beads per mL, stable in aqueous solutions, methanol, ethanol and DMSO). The water was replaced with buffer A (10 mM Tris pH 7.4 and 250 mM NaCl) by centrifugation (13 000 rpm for 2 min) followed by removal of the supernatant and resuspending the beads in an equal volume of buffer A. For the immobilization of the His-tagged proteins (BcLOV4-mCherry, VVDHigh, iLID & Nano, and nMagHigh & pMagHigh), 1 μM of His-tagged protein was incubated with 5 mg per mL of beads in buffer A for 1 h at 4 °C. The excessive protein was removed by centrifugation, and removal of the supernatant and resuspension in buffer A was performed twice. Before each experiment, the functionalized beads were dispersed in solution by sonication for 2 min.

For bead aggregation experiments with heterodimerizing protein pairs (iLID & Nano or nMagHigh & pMagHigh), 25 μL of each bead type (5 mg mL^−1^) were mixed. For homodimerizing proteins (BcLOV4-mCherry or VVDHigh), 50 μL of beads (5 mg mL^−1^) were used. The 50 μL of beads were diluted to a total volume of 300 μL in buffer A and either kept under blue illumination (0.5 mW cm^−2^, LED blue light panel, Albrillo LL-GL003, 225 LEDs, 460 nm, and 14 W), in the dark or in the presence of the chemiluminescence reaction (0.6 U per mL HRP, 200 μM luminol and 500 μM H_2_O_2_) in LoBind Eppendorf tubes under gentle agitation on an orbital shaker at 50 rpm. Subsequently, the samples were fixed at defined time points with 300 μL of 10% (w/v) paraformaldehyde (PFA) for 20 min. 300 μL of each sample was transferred into an 8 well imaging chamber (Lab-Tek®) with a cut pipette tip and the beads were allowed to settle for 30 min. Bead aggregation at different time points was measured by preparing parallel samples that were fixed at different time points. Bright field images were acquired on an inverted fluorescent microscope (DMi8, Leica) through a 40× air objective and for each sample 15 images (130 μm × 130 μm) with a total area of 0.25 mm^2^ were acquired. Each experiment was performed in triplicate. All images were processed with ImageJ 1.64 and the aggregation ratio was analysed as previously described.^32^

### GUV preparation

GUVs were prepared with the gel assisted rehydration method as described before.^33^ The lipid composition of GUVs for the recruitment of Nano-mOrange to iLID functionalized GUVs was 10 mg per mL POPC + 2 mol% biotin-PE + 1 mol% DiD dye and for the recruitment of BcLOV4-mCherry it was 10 mg per mL POPC + 30 mol% POPG + 0.5 mol% biotin-PE + 1 mol% DiD dye. In all other experiments the GUVs were prepare with 10 mg per mL POPC. During the film rehydration, buffer B (10 mM Tris pH 7.4 and 100 mM NaCl) with 100 mM sucrose and 0.6 U per mL HRP was used. 800 μL GUVs were harvested after 1 h rehydration at room temperature and 400 μL of the solution was transferred into a Protein LoBind® tube. Then, GUVs were washed three times with 1 mL of buffer B containing 100 mM glucose by centrifuging at 1000 rpm for 10 min and removing the top 1 mL of the buffer solution. Finally, 200 μL GUV solution from the bottom of the tubes was harvested.

### Luminescence microscopy

Images were obtained with a Luminoview LV200 (Olympus) bioluminescence imaging system, equipped with an EM-CCD camera (0.688 MHz EM-CCD CAM-ImageEM X2, Hamamatsu, Japan). GUVs were prepared as described above with POPC, 1% DiD and 0.6 U per mL encapsulate HRP. 50 μL of GUV solution was added into an 18-well ibidi glass bottom μ-slide, which was precoated with 3% w/v BSA. After addition of 200 μM luminol and 500 μM H_2_O_2_ and the position the GUVs was determined in the bright field channel. The GUVs were monitored in the chemiluminescence and DiD channels, where the bioluminescence was monitored using a 400–500 nm filter and an exposure time of 10 min with an EM gain of 400, using a UPLSAPO 60× Apochromat objective, followed by 10 ms exposure to image the membrane dye DiD. This imaging sequence was repeated three times to measure the chemiluminescence between 0–10, 10–20 and 20–30 min.

### Protein recruitment to GUVs

To avoid the movement of GUVs upon addition of different reagents under the microscope, the GUVs were immobilized on a supported lipid bilayer (SLB) through biotin–streptavidin interaction. Small unilamellar vesicles (SUVs) were prepared by drying 100 μL 10 mg per mL DOPC + 0.5 mol% biotin-PE in a glass flask, rehydrating it with 1 mL buffer B and sonicating the solution for 15 min until clear. The surface of 18 well μ-slide dishes was activated with 150 μL of 2 M NaOH solution for 1 h and subsequently washed with excess MilliQ water. 15 μL SUVs (1 mg per mL lipid) was mixed with 150 μL buffer B containing 10 mM CaCl_2_ and added into each well and incubated for at least 20 min for SLB formation. The SLBs were washed with excess buffer B without ever dying the well. Then, the SLB was incubated with 1 μM streptavidin for 30 min and the excess was removed through washing with excess buffer B. Finally, 50 μL GUVs with biotin-PE in the membrane were added on top and incubated for 10 min such that GUVs bound to the SLB through the biotin–streptavidin–biotin linker.

### Chemiluminescence triggered protein recruitment to GUVs

For the recruitment of Nano-mOrange, 5 μM biotinylated iLID was added into the chamber with the GUVs, the sample was incubated for 30 min in the dark and the excess of iLID was washed with excess buffer B. Then, 200 nM Nano-mOrange was added and incubated for 10 min. Subsequently, 100–200 μM luminol and 250–500 μM H_2_O_2_ were added to the sample and the recruitment of the protein was followed by confocal microscopy. As a positive control, the chemiluminescence reagents were left out and half of a GUV was illuminated using the fluorescence recovery after photobleaching (FRAP) option with a 488 nm laser (1% intensity) and synchronous imaging.

For the recruitment of BcLOV4-mCherry, 200 nM was added to the GUVs and the experiment was carried out as described above.

All images were acquired on a Leica SP8 confocal laser scanning microscope through a 63× water objective equipped with four laser lines (405 nm, 488 nm, 552 nm and 638 nm), mCherry and mOrange (*λ*_Ex_ = 552 nm and *λ*_Em_ = 560–600 nm), and DiD dye (*λ*_Ex_ = 638 nm and *λ*_Em_ = 650–700 nm).

For the analysis of Nano-mOrange and BcLOV4-mCherry recruitment to GUV membranes, the background of each image was subtracted. A mask of the membrane was generated using the DiD channel with a Gaussian blur (sigma radius 50). The mOrange and mCherry fluorescence in the masked area was measured over time, plotted using OriginPro 9.1 and fitted with a smoothen function using the Savitzky–Golay method (point of window: 50, boundary condition: none, and polynomial order: 2). The data from the first 5 min were not analysed due to fluctuation in the images after addition of chemiluminescence reagents.

### Cell culture

HL 60 cells were a kind gift from R. Hallmann/L. Sorokin. HL 60 cells were cultured in RPMI 1640 with 2 mM glutamine and 10–20% FBS at 37 °C and 5% CO_2_. 5 × 10^5^ cells were seeded into a T25 flask with different concentrations of DMSO for differentiation for 1 to 4 days (1.25% v/v DMSO for 1 day if not specified otherwise). Subsequently, the cells were harvested by centrifugation at 290*g* for 10 min, followed by washing with and resuspending in 3 mL PBS supplemented with 5 mM glucose and 1 mM CaCl_2_. The cells were stained using Vybrant™ DiD cell-labeling solution following the manufacturer's protocol one day before the experiment.

### Fluorescence flow cytometry analysis for the validation of HL 60 cell differentiation

10^6^ HL 60 or dHL 60 were harvested through centrifugation at 290*g* for 6 min at 4 °C and washed with PBS. The cells were resuspended in 50 μL PBS and incubated with 10 μL (1 : 2 dilution) monoclonal antibody: PE labeled IgG1 mouse anti human CD11b (eBioscience 12-0018) for 30 min on ice. The pellet was washed twice with PBS and resuspended with 1 mL PBS. The cells were analysed with flow cytometry (Invitrogen, Attune NxT Flow Cytometer) with at least 1 × 10^4^ gated events. A PE-labelled isotype-specific monoclonal antibody (eBioscience 12-4714) was used as the negative control.

### Chemiluminescence measurement from dHL 60 cells

Measurements were performed on a multimode plate reader (Spark, Tecan Life Science) with 400–500 nm using sterilized white bottom white plates that were coated with 3% BSA for 10 min. 5 × 10^3^ dHL 60 cells in 200 μL PBS with 5 mM glucose, 1 mM CaCl_2_, 1 mM NaN_3_, 10 μM L-012, and different concentrations of HRP were added to each well. Respiratory bursts were stimulated with 1 μM PMA and chemiluminescence was measured immediately afterwards for 90 min. Luminescence outputs were reported as the integral under each curve.

### BcLOV4-mCherry recruitment to the dHL 60 cell by chemiluminescence

1 × 10^3^ dHL 60 cells in 100 μL buffer were added into 18-well ibidi glass bottom μ-slides, which were precoated with 3% w/v BSA. Subsequently, 50 nM BcLOV4-mCherry and reagents for chemiluminescence (10 μM L-012, 2 U per mL HRP, NaN_3_ and PMA) were added into the cells. The recruitment of BcLOV4-mCherry was monitored by confocal microscopy as described above. As a positive control, the chemiluminescence reagents were left out and the cells were illuminated using the fluorescence recovery after photobleaching (FRAP) option with a 488 nm laser (0.5% intensity) and synchronous imaging.

For the analysis of BcLOV4-mCherry recruitment to the dHL 60 membrane, the background of each image was subtracted. A mask of the membrane was generated using the DiD channel with a Gaussian blur (sigma radius 50). The mCherry fluorescence in the masked area was measured over time and averaged from three independent cells with independent duplication, plotted using OriginPro 9.1. The scale bar represents standard error.

### Statistical analysis

All images were processed with ImageJ 1.64. For the bead aggregation assay, the data were plotted as mean ± SEM from technical triplicates performed on different days. The statistical significance was determined by one-way ANOVA with OriginPro 9.1. *p* values: ns > 0.05, **p < 0.1, and ***p < 0.01.

## Author contributions

Y. J., A. H., B. N. M. and S. V. W. designed the experiments, A. H. established BcLOV4-mCherry binding to GUVs and Y. J. conducted all other experiments. B. N. supported Y. J. with cell culture. All authors have given approval to the final version of the manuscript.

## Conflicts of interest

There are no conflicts to declare.

## Supplementary Material

SC-015-D3SC04815B-s001

SC-015-D3SC04815B-s002

SC-015-D3SC04815B-s003

SC-015-D3SC04815B-s004

## Data Availability

The datasets generated during the current study are available from the authors on reasonable request.
