# Supplementary material for: Photoactivation of LOV domains with chemiluminescence
Source: Chem Sci. 2023 Dec 11;15(3):1027–38. doi: 10.1039/d3sc04815b (PMC10793642; doi:10.1039/d3sc04815b)
Supplement: SC-015-D3SC04815B-s001 [file SC-015-D3SC04815B-s001.pdf]

Electronic Supplementary Material (ESI) for Chemical Science.
This journal is © The Royal Society of Chemistry 2023

Electronic Supporting Information

Photoactivation of LOV domains with chemiluminescence

Yuhao Ji,[a] Ali Heidari,[a] Brice Nzigou Mombo,[a] and Seraphine V. Wegner[a,]*

a. Institute of Physiological Chemistry and Pathobiochemistry, University of Münster, 48149 Münster, Germany.

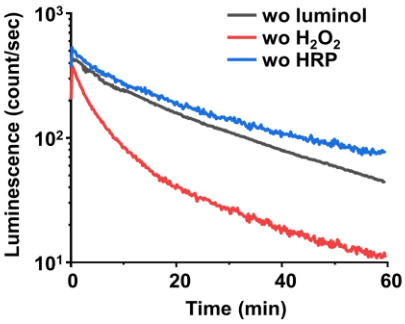

Figure S1 Chemiluminescence production efficiency background. Experiment in Fig. 1c was repeated without any single component of chemiluminescence (200 µM luminol, 500 µM H_2O_2 or 0.6 U/ml HRP).

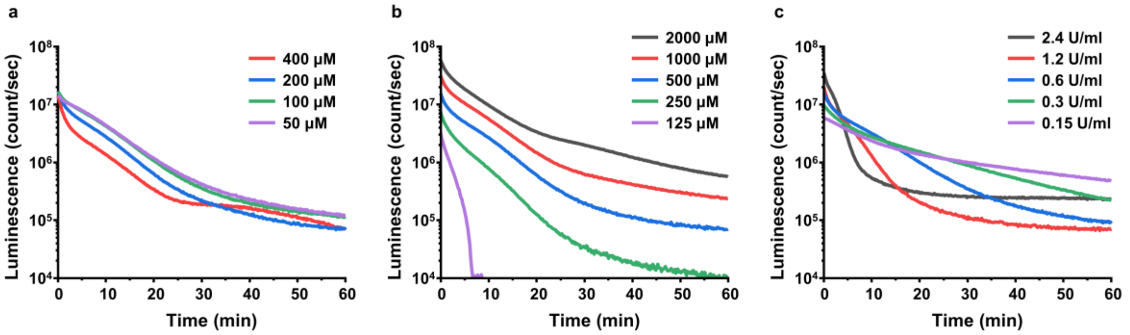

Figure S2 Controlling chemiluminescence efficiency and kinetics by altering concentration of chemiluminescence reagents. **(a)** Titration of luminol, with 500 µM H_2O_2 and 0.6/ml HRP. **(b)** Titration of H_2O_2, with 200 µM luminol and 0.6 U/ml HRP. **(c)** Titration of HRP, with 200 µM luminol and 500 µM H_2O_2. Chemiluminescence signals were shown in the range between 10^4 to 10^8 count/sec for comparison.

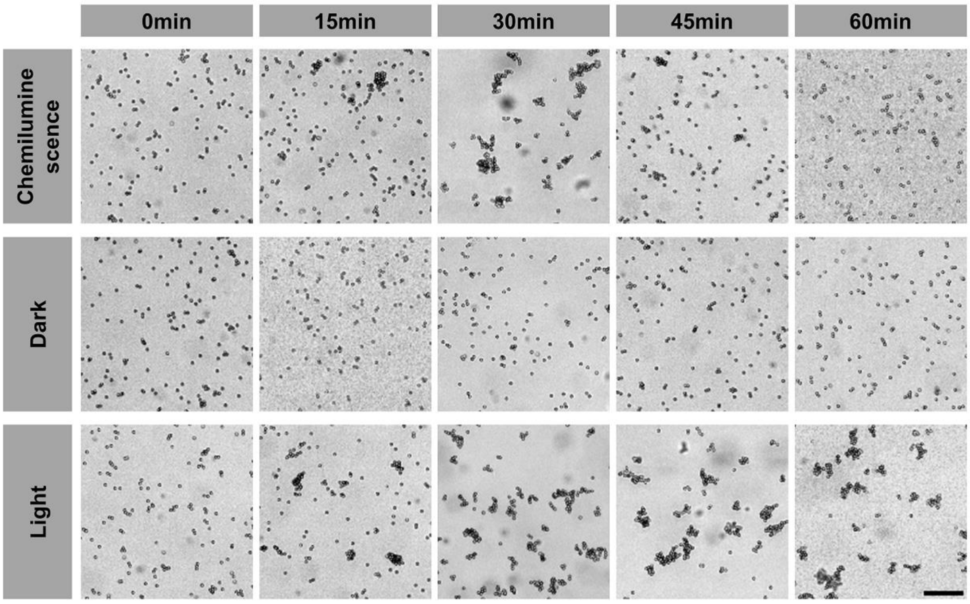

Figure S3 BcLOV4-mCherry functionalized polystyrene bead aggregation under chemiluminescence (200 μM luminol, 500 μM H_2O_2 and 0.6 U/ml HRP), in the dark (negative control) and under continuous blue illumination (positive control). Scale bar is 30 μm.

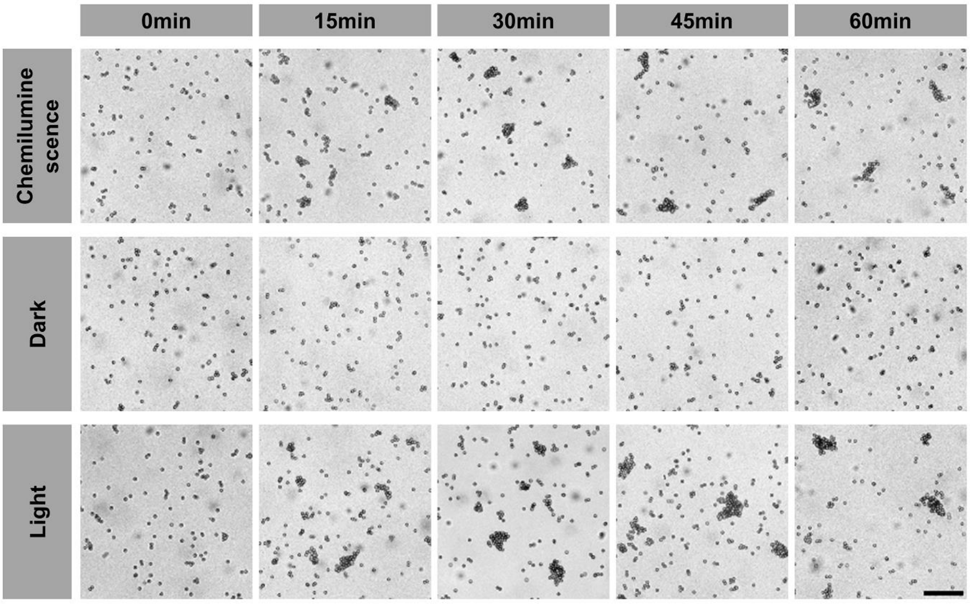

Figure S4 VVDHigh functionalized polystyrene bead aggregation under chemiluminescence (200 µM luminol, 500 µM H_2O_2 and 0.6 U/ml HRP), in the dark (negative control) and under continuous blue illumination (positive control). Scale bar is 30 µm.

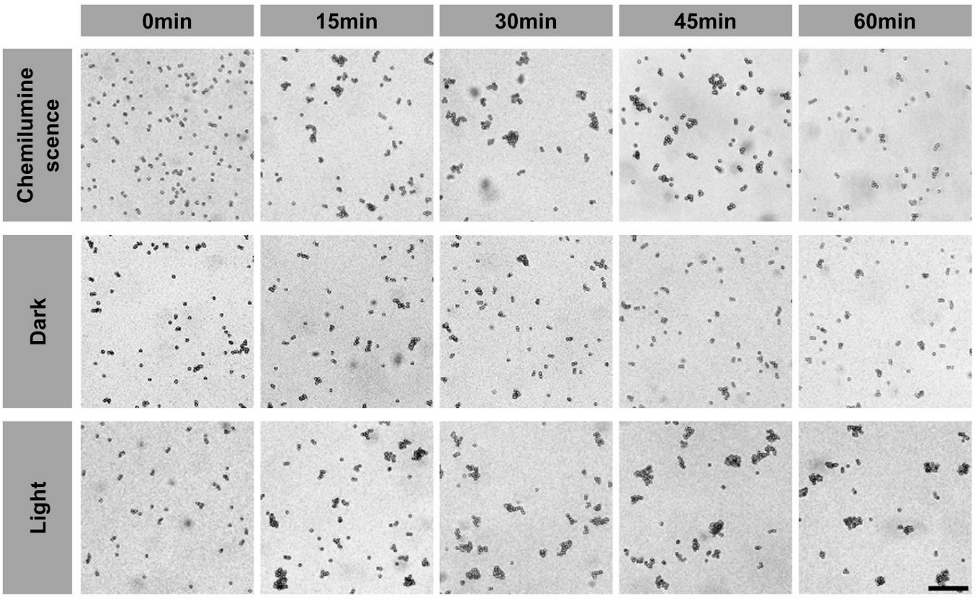

Figure S5 iLID and Nano (mixed in 1:1 ration) functionalized polystyrene bead aggregation under chemiluminescence (200 µM luminol, 500 µM H_2O_2 and 0.6 U/ml HRP), in the dark (negative control) and under continuous blue illumination (positive control). Scale bar is 30 µm.

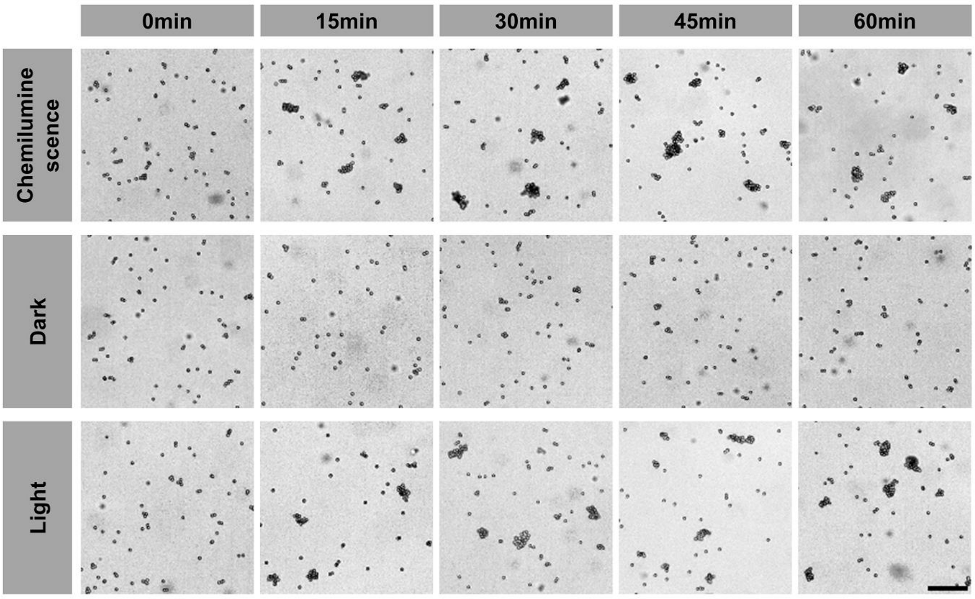

Figure S6 nMagHigh and pMagHigh (mixed in 1:1 ration) functionalized polystyrene bead aggregation under chemiluminescence (200 µM luminol, 500 µM H_2O_2 and 0.6 U/ml HRP), in the dark (negative control) and under continuous blue illumination (positive control). Scale bar is 30 µm.

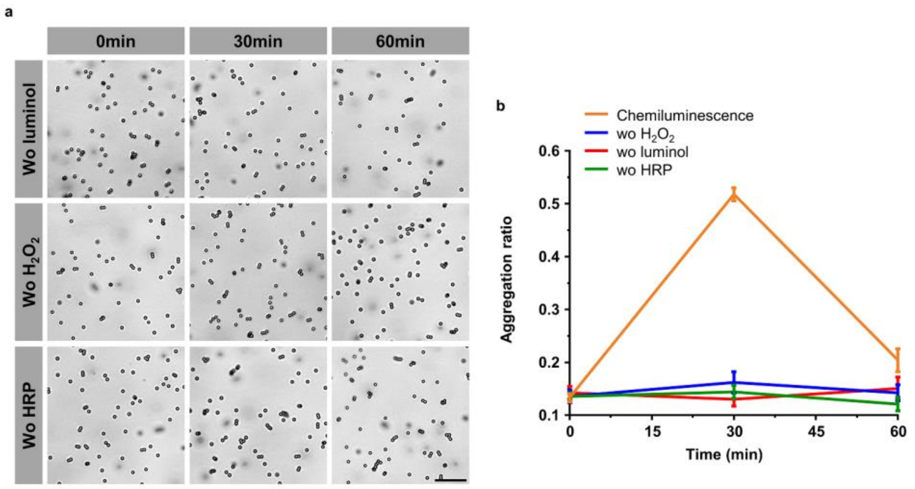

Figure S7 Chemiluminescence reagent influence on BcLOV4-mCherry-coated beads aggregation. Experiment in Fig. 2c was repeated without any one of the chemiluminescence reagent. **(a)** Aggregation of 2 µm polystyrene beads coated with BcLOV4-mCherry without any one of chemiluminescence reaction (200 µM luminol, 500 µM H_2O_2, 0.6 U/ml HRP) at different time points. Scale bar is 30 µm. **(b)** Aggregation ratios of beads functionalized with BcLOV4-mCherry under each control condition, with full chemiluminescence reaction in Fig. 2c for comparison.

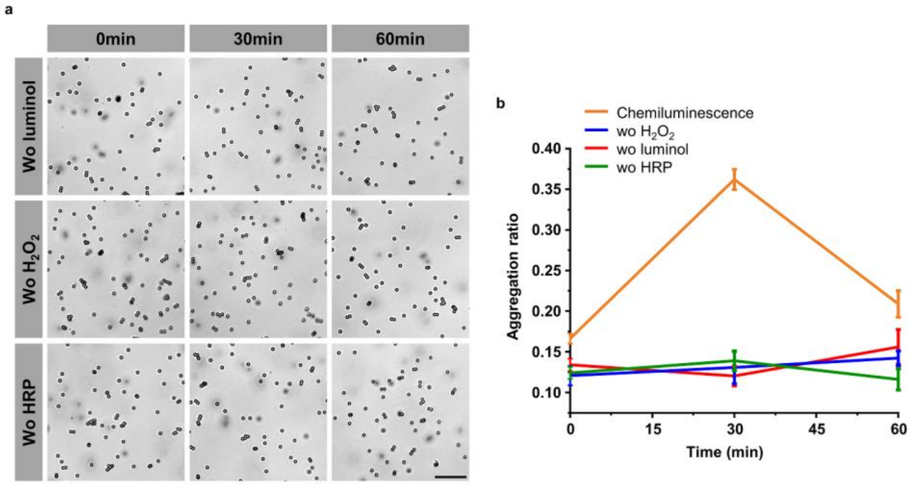

Figure S8 Chemiluminescence reagent influence on iLID & Nano -coated beads aggregation. Experiment in Fig. 2e was repeated without any one of the chemiluminescence reagent. **(a)** Aggregation of 2 µm polystyrene beads coated with BcLOV4-mCherry without any one of chemiluminescence reaction (200 µM luminol, 500 µM H_2O_2, 0.6 U/ml HRP) at different time points. Scale bar is 30 µm. **(b)** Aggregation ratios of beads functionalized with iLID & Nano under each control condition, with full chemiluminescence reaction in Fig. 2e for comparison.

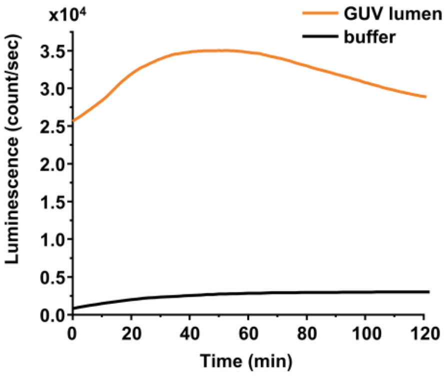

Figure S9 Intracellular chemiluminescence production from GUV. GUVs were loaded with 0.6 U/mL HRP and 200 µM luminol and 500 µM H_2O_2 were added externally. As buffer the washing solution outside the GUVs was used. The chemiluminescence was measured using plate reader with a filter between 400-500 nm.

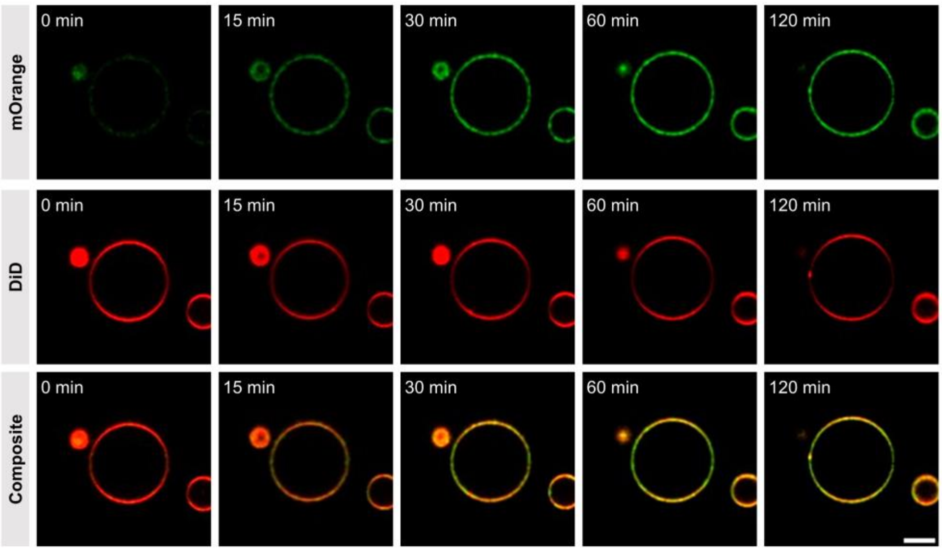

Figure S10 Nano-mOrange (shown in green) recruitment on an iLID functionalized GUV (membrane dye DiD shown in red) by chemiluminescence (200 µM luminol, 500 µM H_2O_2 and 0.6 U/ml HRP). Scale bar is 10 µm. Also see Movie 1.

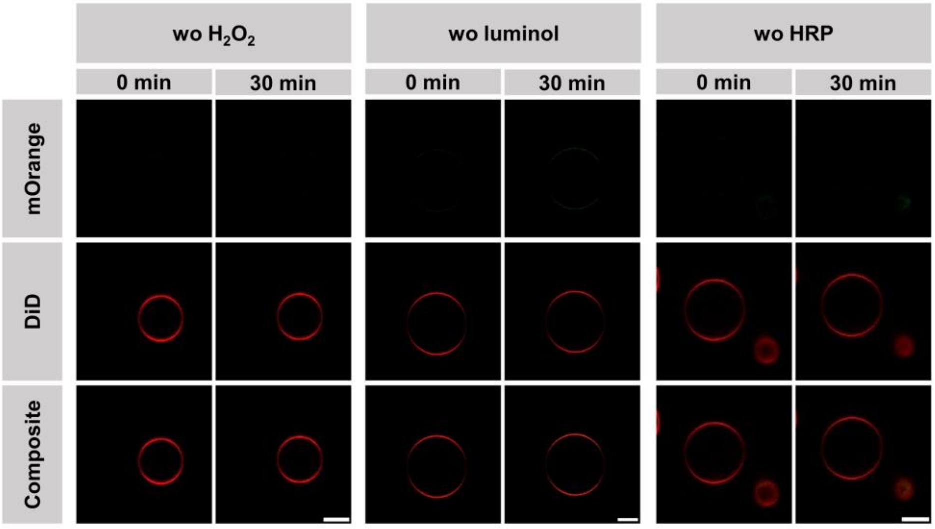

Figure S11 Nano-mOrange (shown in green) recruitment on an iLID functionalized GUV (membrane dye DiD shown in red) in the absence of chemiluminescence (200 µM luminol, 500 µM H_2O_2 and 0.6 U/ml HRP). Scale bar is 10 µm.

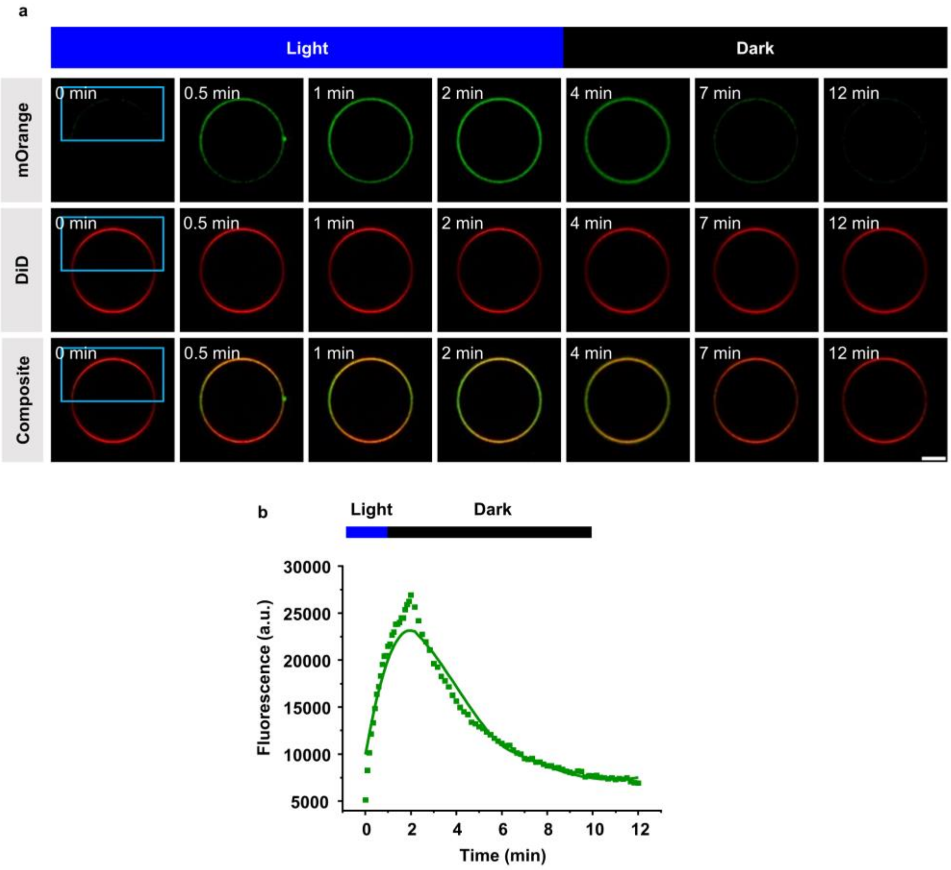

Figure S12 (a) Nano-mOrange (shown in green) recruitment on an iLID functionalized GUV (membrane dye DiD shown in red) with local illumination (shown as blue box) and dissociation in the dark. Scale bar is 10 µm. **(b)** Dynamics of Nano-mOrange recruitment with blue light and dissociation in the dark for experiment in (a).

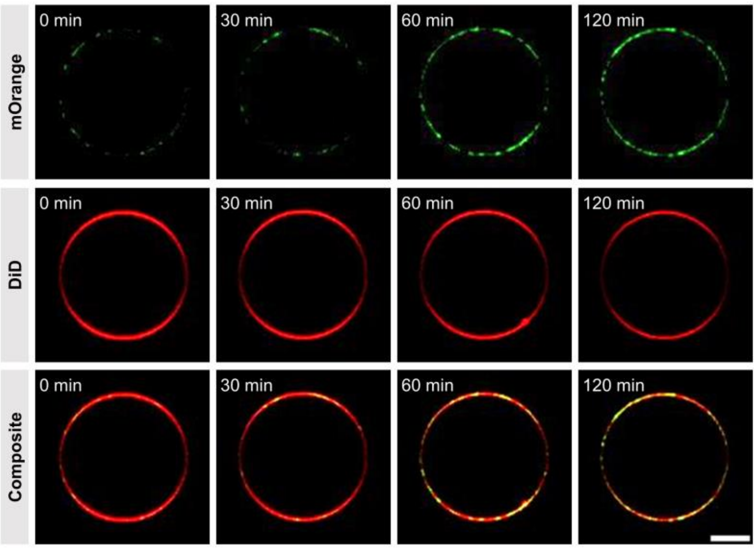

Figure S13 Nano-mOrange (shown in green) recruitment on an iLID functionalized GUV (membrane dye DiD shown in red) by chemiluminescence with lower luminol concentration (100 µM luminol, 500 µM H_2O_2 and 0.6 U/ml HRP). Scale bar is 10 µm.

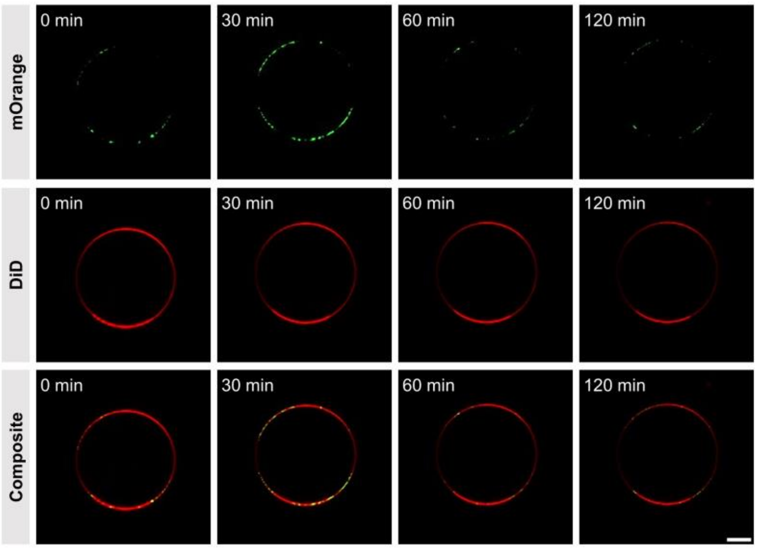

Figure S14 Nano-mOrange (shown in green) recruitment on an iLID functionalized GUV (membrane dye DiD shown in red) by chemiluminescence with lower H_2O_2 concentration (200 µM luminol, 250 µM H_2O_2 and 0.6 U/ml HRP). Scale bar is 10 µm.

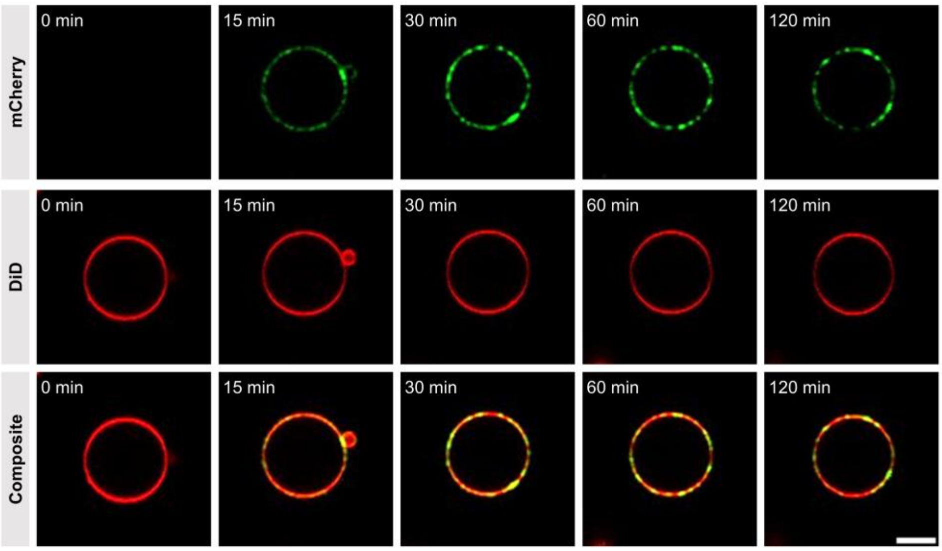

Figure S15 BcLOV4-mCherry (shown in green) recruitment on a negatively charged GUV (membrane dye DiD shown in red) by chemiluminescence (200 µM luminol, 500 µM H_2O_2 and 0.6 U/ml HRP). Scale bar is 10 µm. Also see Movie 2.

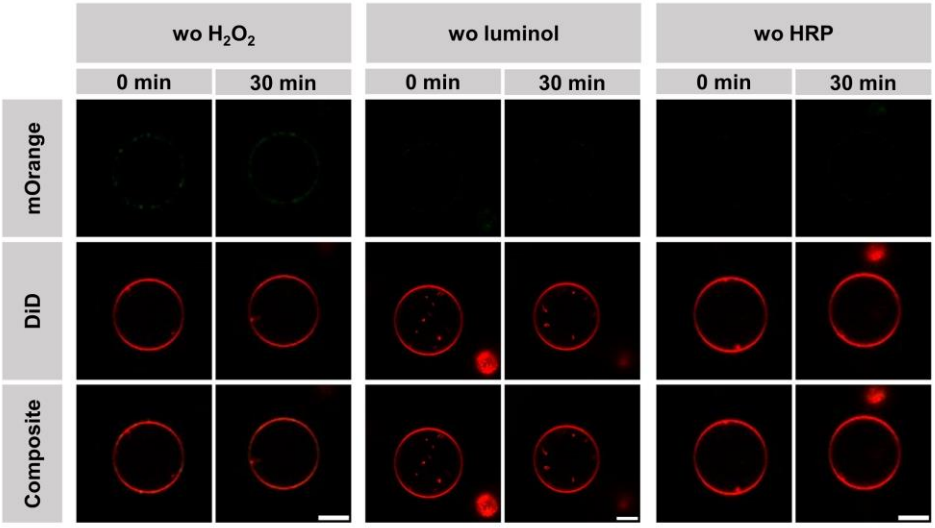

Figure S16 BcLOV4-mCherry (shown in green) recruitment on a negatively charged GUV (membrane dye DiD shown in red) in the absence of chemiluminescence (200 µM luminol, 500 µM H_2O_2 and 0.6 U/ml HRP). Scale bar is 10 µm.

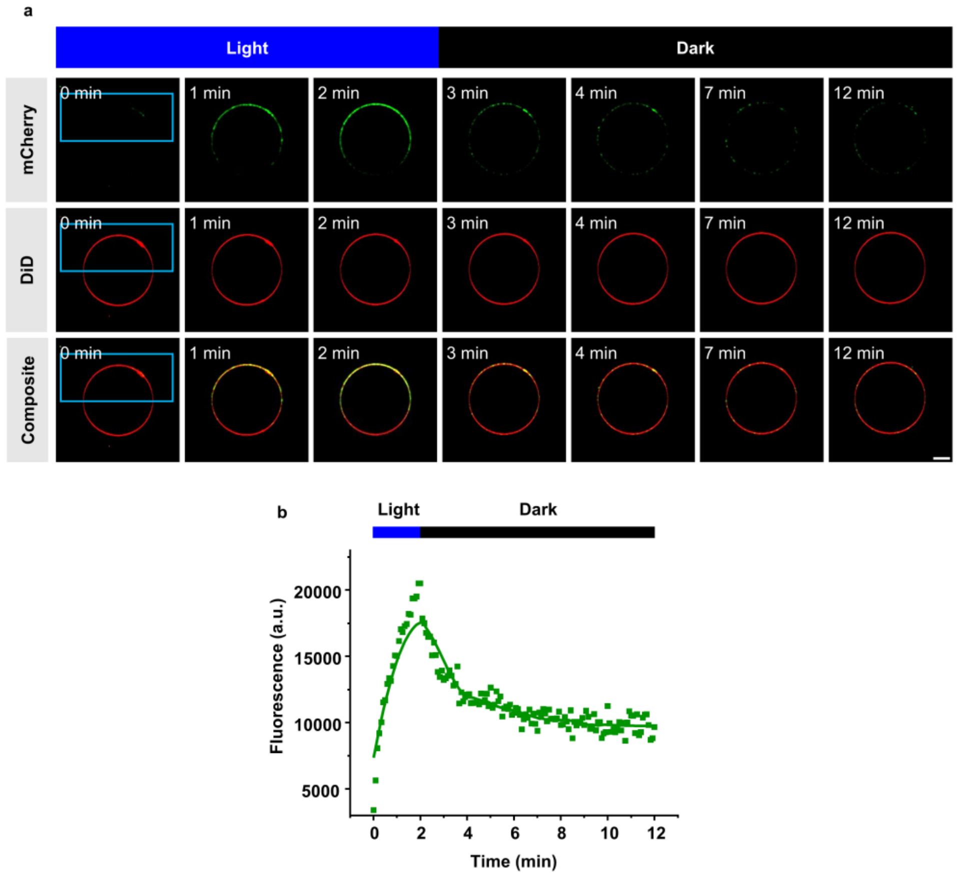

Figure S17 (a) BcLOV4-mCherry (shown in green) recruitment on a negatively charged GUV (membrane dye DiD shown in red) with local illumination (shown as blue box) and dissociation in the dark. Scale bar is 10 µm. **(b)** Dynamics of BcLOV4-mCherry recruitment with blue light and dissociation in the dark for experiment in (a).

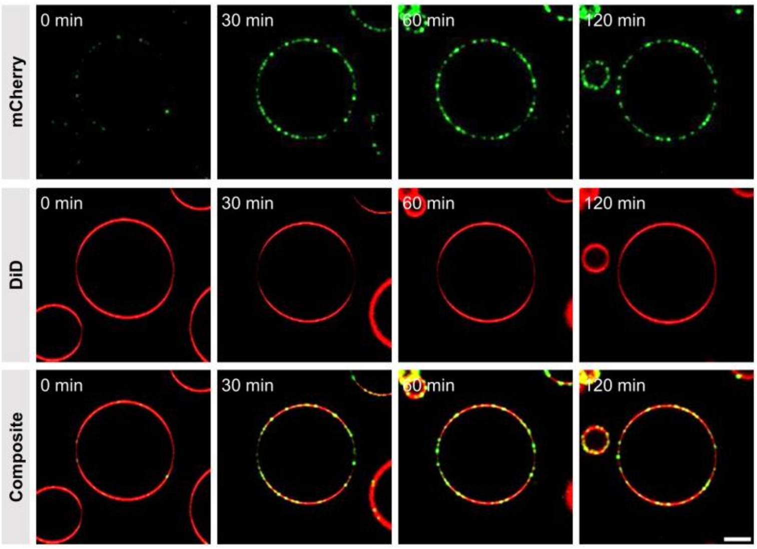

Figure S18 BcLOV4-mCherry (shown in green) recruitment on a negatively charged GUV (membrane dye DiD shown in red) by chemiluminescence with lower luminol concentration (100 µM luminol, 500 µM H_2O_2 and 0.6 U/ml HRP). Scale bar is 10 µm.

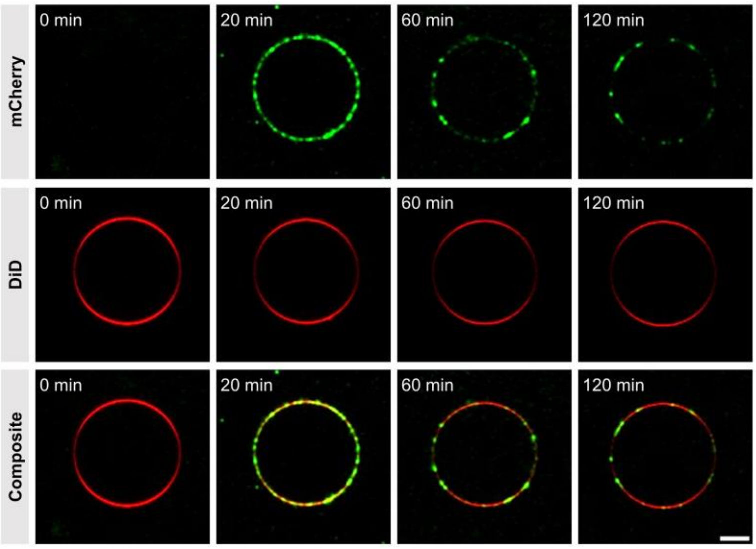

Figure S19 BcLOV4-mCherry (shown in green) recruitment on a negatively charged GUV (membrane dye DiD shown in red) by chemiluminescence with lower H_2O_2 concentration (200 µM luminol, 250 µM H_2O_2 and 0.6 U/ml HRP). Scale bar is 10 µm.

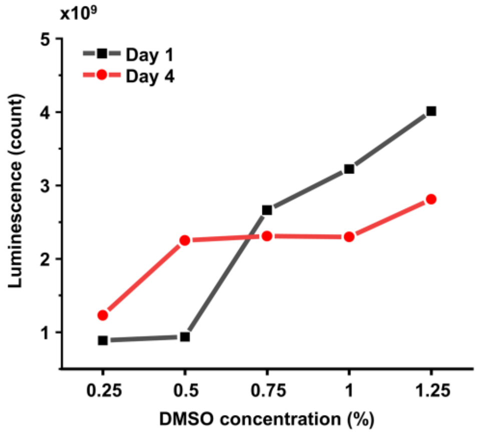

Figure S20 Optimization of HL 60 cell differentiation for chemiluminescence production. dHL 60 cells induced with different concentrations of DMSO were incubated with 1 mM NaN_3 and stimulated with 1 µM PMA. Chemiluminescence were generated from 10 µM L-012 and 2 U/ml HRP. Luminescence output were calculated as the integral during the period of 0-90 min.

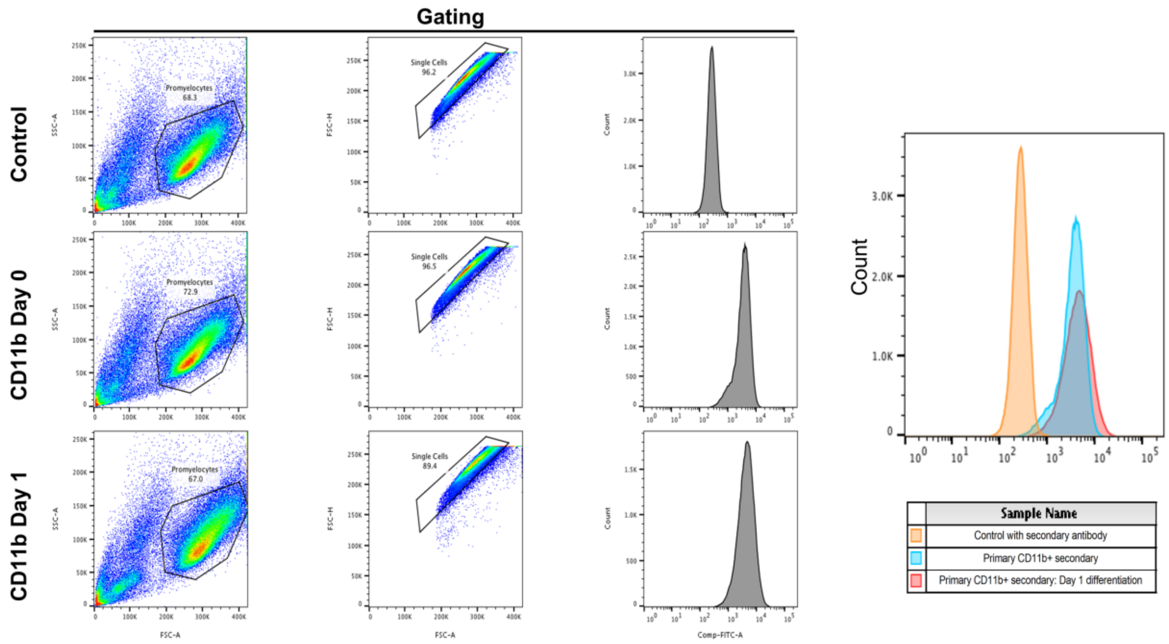

Figure S21 Fluorescence flow cytometry analysis of HL 60 cell differentiation. Differentiation was induced with 1.25 % v/v DMSO for 1 day. Bound anti-CD11b antibody from the total gate was evaluated as an indicator.

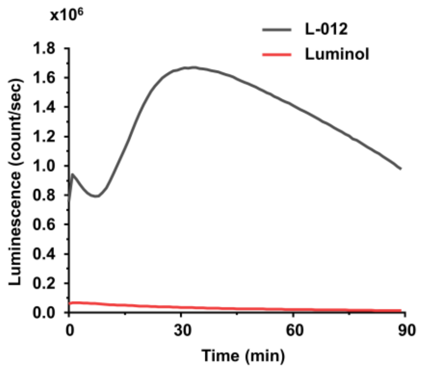

Figure S22 Comparison of chemiluminescence generation from luminol derivative L-012 and luminol. 10 μM L-012 or luminol was mixed with 10 mU/ml HRP and 200 μM H_2O_2 in PBS.

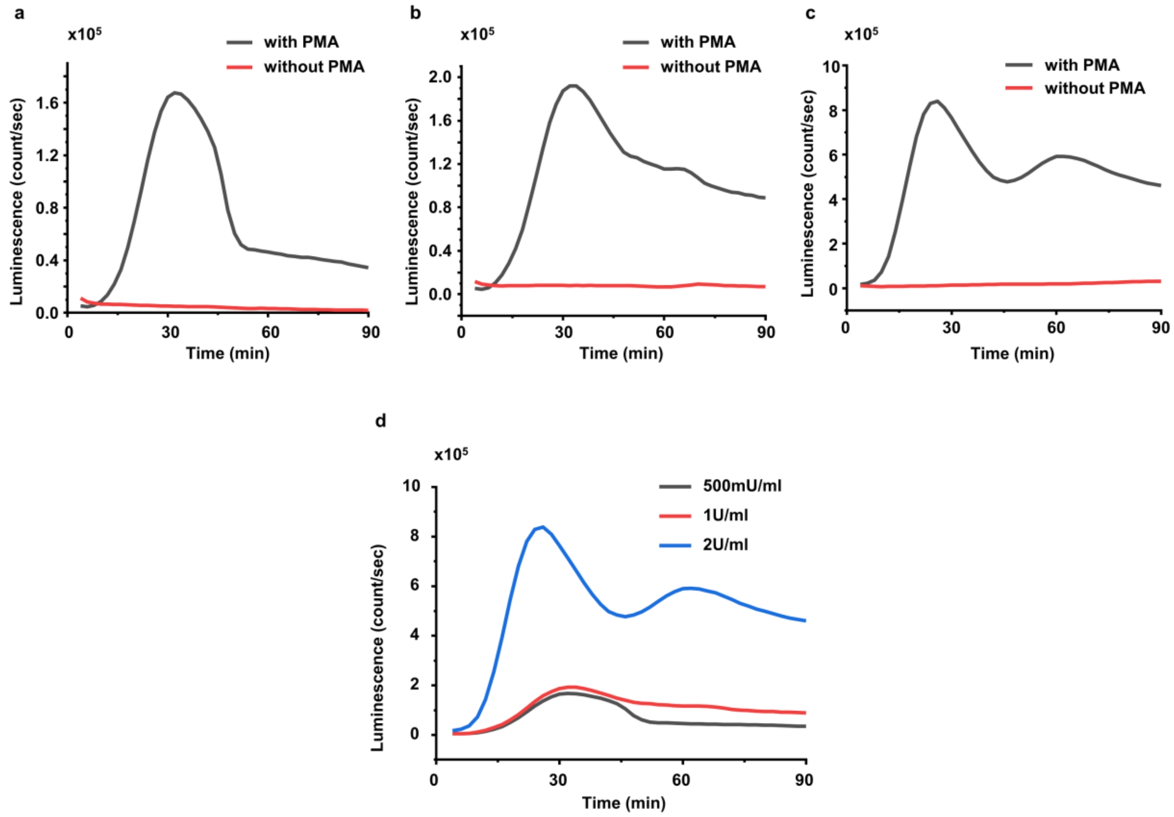

Figure S23 Chemiluminescence generation from dHL 60 cells respiratory burst stimulated with PMA, and optimization of HRP concentration. Respiratory burst from dHL 60 cell (1.25 % v/v incubation with DMSO for 1 day) was initiated with 1 mM NaN_3 and 1 µM PMA. Chemiluminescence were generated from 10 µM L-012 and 500 mU/ml **(a)**, 1 U/ml **(b)**, and 2 U/ml **(c)** HRP. **(d)** Comparison of chemiluminescence production from PMA-stimulated dHL 60 cells with different HRP concentration.

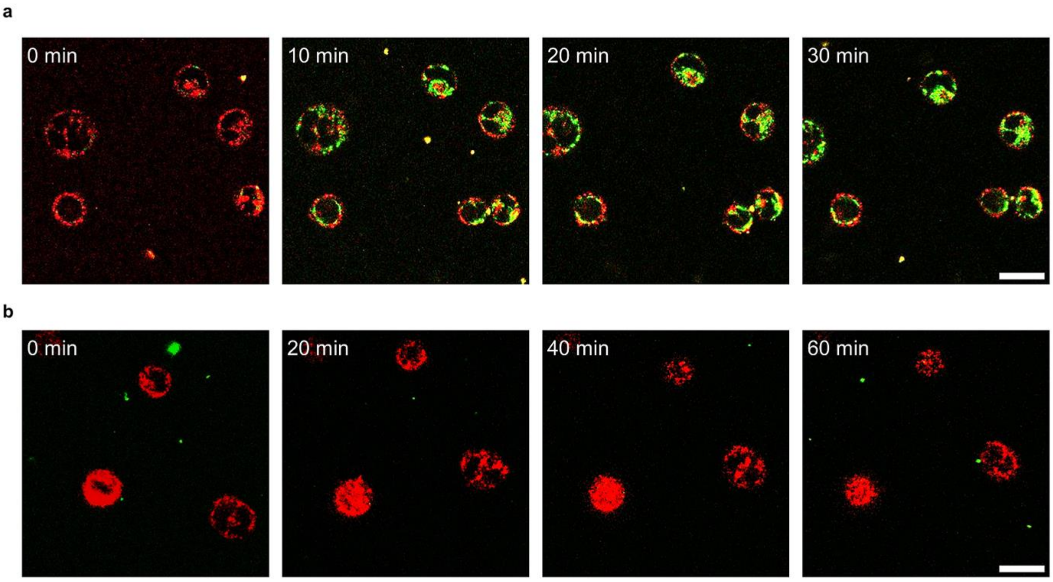

Figure S24 BcLOV4-mCherry recruitment on dHL 60 cell membrane under external blue illumination (0.5 % 488 nm laser) **(a)** and dark **(b)** as positive and negative controls, respectively.

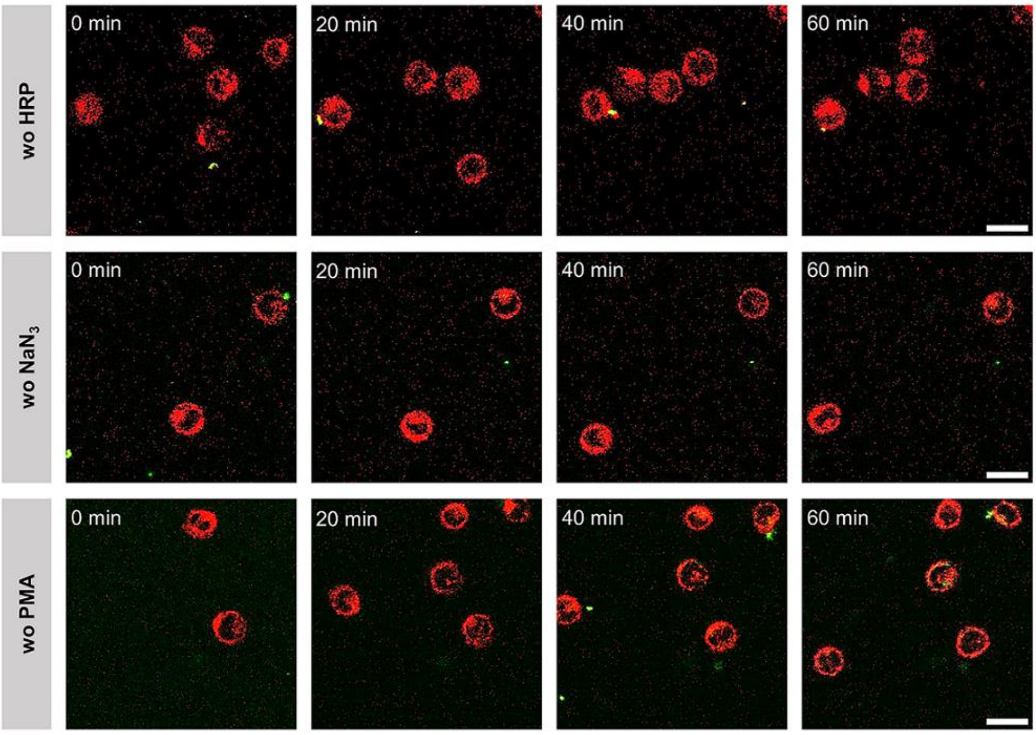

Figure S25 Controls for BcLOV4-mCherry recruitment on dHL 60 cell membrane by chemiluminescence. Experiment in Fig. 6a was repeated without any one of the reagents (2 U/ml HRP, 1 mM NaN_3 or 1 µM PMA).
